# Post-cesarean section laparoscopic cholecystectomy: a case report

**DOI:** 10.1186/s12884-023-05767-3

**Published:** 2023-06-17

**Authors:** Sarah Magdy Abdelmohsen, Mohamed Mahmoud Zidan, Sherif Salah Eldeen Fahmy, Ahmed Saleh Baghdady

**Affiliations:** 1 Department of Pediatric Surgery, Aswan University Hospital, Aswan University, Aswan City, Egypt; 2 Department of General Surgery, Aswan University Hospital, Aswan university, Aswan City, Egypt; 3 Department of Obstetrics and Gynecology, Aswan University Hospital, Aswan University, Aswan City, Egypt

**Keywords:** Anencephaly, Acute cholecystitis, Surgical emergency during pregnancy, Combined approaches, Cesarean section

## Abstract

**Background:**

Laparoscopic cholecystectomy at the time of cesarean section is novel in medicine. It is safe, feasible, and cost-effective.

**Case presentation:**

A 29-year-old G3P2 + 0 woman had two previous cesarean sections. She was pregnant at 32 weeks. The fetus had anencephaly. She had acute cholecystitis. Laparoscopic cholecystectomy done at the time of termination of pregnancy by cesarean section.

**Conclusions:**

In a critical period, such as acute cholecystitis, the combination of laparoscopic cholecystectomy immediately post cesarean section is effective if the surgeon is highly qualified and experienced.

## Background

The second most frequent surgical emergency during pregnancy, behind appendicitis, is acute cholecystitis [1]. Obstetric ultrasonography detects gallstones in 2–4% of pregnant women, while symptomatic cholelithiasis and cholecystitis during pregnancy only happen in 5–10 of every 10,000 gestational women [2]. Concurrent cholecystectomies at the time of cesarean section is cost-effective because it avoids rehospitalization and frequent exposure to anesthesia [2, 3]. Herein, the author reports that a pregnant woman complained of acute cholecystitis, and at the same sitting, she had a laparoscopic cholecystectomy accompanied by a lower segment cesarean section. To the best of our knowledge, this is the second reported laparoscopic cholecystectomy post-cesarean due to acute cholecystitis. Sánchez et al. (2011) described the first combined approach to treating acute cholecystitis [1].

## Case presentation

A 29-year-old G3P2 + 0 woman with two previous cesarean sections at 32 weeks of gestation. The woman was informed that the fetus had a congenital anomaly (anencephaly) that wasn’t suitable for life in addition to polyhydramnios. Because of her previous two cesarean sections, she was prepared to terminate her pregnancy via elective cesarean section. Then a woman developed pain in the right upper quadrant and epigastric areas for 24 h. She had nausea and vomiting but no fever or jaundice.

Physical examination refers to a gravid uterus large for dates by 4 weeks. She had tenderness on palpation in the right upper quadrant and epigastric areas referred to the tip of the right shoulder with a positive Murphy’s sign. WBCs were 13,000 nmol/mm3, and normal results from other tests such as amylase, liver, and kidney function tests were found in the laboratory. Abdominal ultrasonography confirmed an anencephalic fetus at 32 ± 1 weeks’ gestation, cholelithiasis and pericholecystic collection, and no biliary tract dilatation.

The symptoms didn’t improve with conservative treatment like hydration, antibiotics, and analgesics for 48 h. So, the decision to perform a laparoscopic cholecystectomy concurrent with an immediate post-cesarian section was taken by an experienced surgeon.

**Fig. 1:**
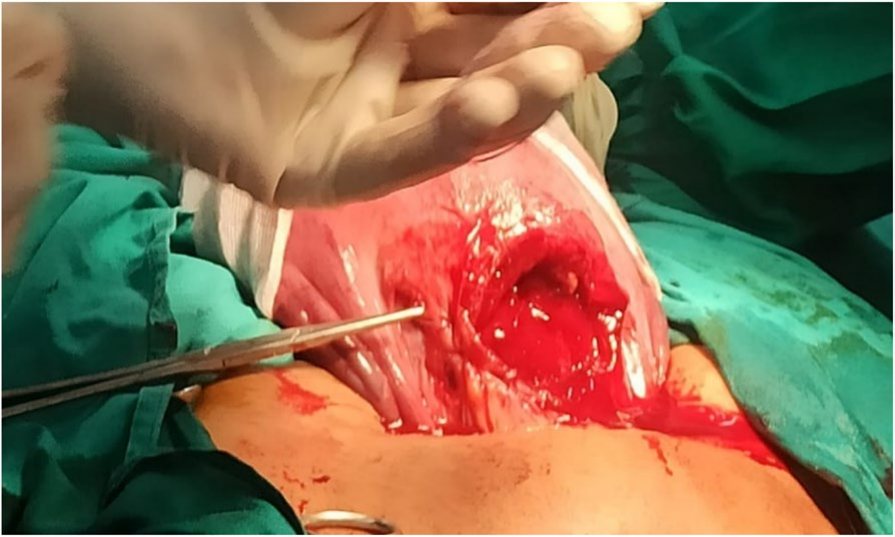
Standard cesarean section done first

Under general endotracheal intubation, the caesarian section was done first through the previous Pfannenstiel incision. Then comes the transverse hysterotomy incision, pregnancy termination, and repair of the uterine and anterior abdominal wall Pfannenstiel incision (the same as a standard cesarean section) (Fig. 1).

**Fig. 2:**
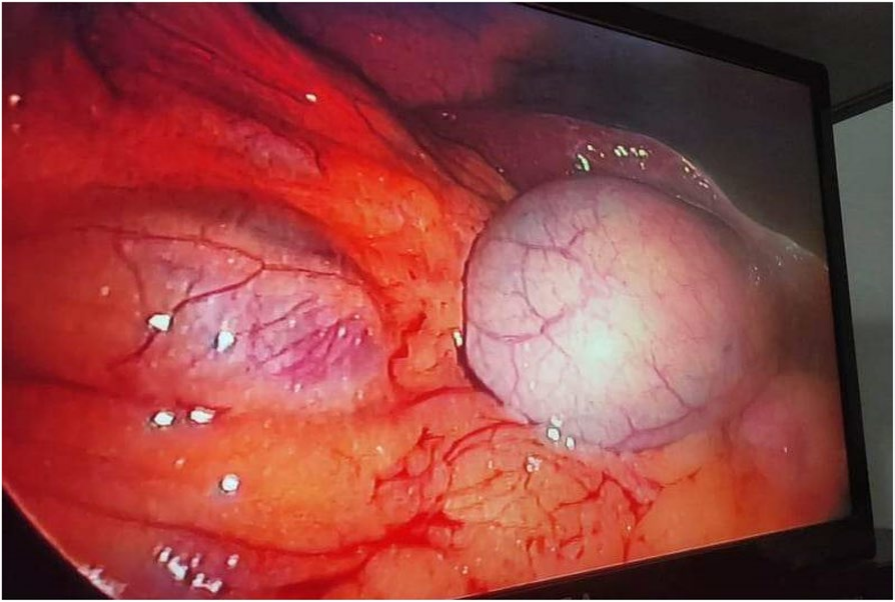
An enlarged congested gall bladder is on the right side attached to the liver. The right colonic flexure appears on the left

A laparoscopic camera port (10 mm) was inserted just supraumbilical; another port canula was on the right upper quadrant at the midclavicular line (5 mm), the third port was in the epigastric area (10 mm); and the last port was at the right anterior axillary line (5 mm). Pneumoperitoneum was established with CO2 at a pressure of 12 mmHg. The camera video showed an enlarged and congested gallbladder (Fig. 2). Dissection of the gallbladder and ligation of the cystic artery and cystic duct were done in the ordinary way with a metal clip without any difficulty. The gallbladder was placed inside a plastic retrieval bag before its withdrawal from the epigastric port site. The sites of the laparoscopic ports were then closed after we were assured that there was no intraabdominal bleeding. The total operating time was 1 h and 35 min.

Follow-up with the patient involves simply visualizing the amount of vaginal bleeding to detect uterine hemorrhage and frequent blood pressure monitoring by the circulating nurse to detect any non-visualized intraabdominal bleeding. The patient continued on good hydration, analgesics, and Ampicillin-Sulbactam 1.5 g every 12 h for 48 h. The antibiotic Ampicillin-Sulbactam 750 mg film-coated tablet was then started every 8 h for 5 days. The patient was discharged on the third postoperative day with a good outcome. The stitches were removed with clean wounds on the tenth postoperative day.

## Discussion and conclusions

Concurrent laparoscopic cholecystectomy with cesarean section is a newly introduced procedure in the surgical field. There are few cases reported in the literature [1, 3–5]. However, Mushtaque et al. (2012) reported that 32 pregnant women were subjected to open cholecystectomy post-cesarean Sect [6]. In 2019, Mushtaque et al. reported that eight women were subjected to laparoscopic cholecystectomy at the time of cesarean Sect [2].

In symptomatic gallbladder disease in pregnancy, considering the short duration of pregnancy, patients should be treated conservatively until there is strong evidence of complications such as acute cholecystitis [2, 7]. Laparoscopic surgery seems to be a safe alternative to open surgery during pregnancy and at the time of a cesarean section. It allows the surgeons to operate through small incisions and reduce the risks of surgical infection, blood loss, and incisional hernia [2, 6–8].

It is important to secure the repair of the anterior abdominal wall Pfannenstiel incision to prevent the occurrence of parietal emphysema resulting from passage of gases through the incision of the cesarean section. We repaired the abdominal wall in layers used continuous sutures like water tight closure. Also, pneumoperitoneum was established at low pressure 12mmgh.

Pelosi et al. (1999) reported hand-assisted laparoscopic cholecystectomy through the cesarean laparotomy incision, which was left open, and, under direct visual and manual guidance, laparoscopic cannulas were placed [3]. This operative technique needs a pneumo-sleeve system for protection of the laparotomy incision from infection.

We recommend a comparison study between laparoscopic, minilaparotomy, and hand-assisted laparoscopic cholecystectomy immediately post-cesarean section. Laparoscopic cholecystectomy immediately after cesarean section in one sitting is safe, cost-effective, has low morbidity, and keeps the benefit of using minimally invasive techniques.

## Data Availability

The datasets used and/or analysed during the current study available from the corresponding author on reasonable request.
